# Navigating maternal health challenges in Bangladesh: an analysis of pregnancy complications and care-seeking behaviours using nationally representative surveys

**DOI:** 10.7189/jogh.16.04261

**Published:** 2026-08-07

**Authors:** Abu Bakkar Siddique, Anindita Saha, Md Moinuddin Haider, Aniqa Tasnim Hossain, Ashiquzzaman, Rifath Binta Modasser, Nuruzzaman Lucky, Afrin Naher Kana, Mahbuba Mehjabin, Shafiqul Ameen, Anisuddin Ahmed, Shams El Arifeen, Ahmed Ehsanur Rahman

**Affiliations:** 1 Maternal and Child Health Division, International Centre for Diarrhoeal Disease Research, Bangladesh (icddr,b), Dhaka, Bangladesh; 2 Department of Women’s and Children’s Health, Global Health and Migration Unit, Uppsala University, Uppsala, Sweden

**Keywords:** maternal complications, preeclampsia, haemorrhage, convulsions, care-seeking, Bangladesh

## Abstract

**Background:**

Bangladesh reports about 6500 maternal deaths annually, mainly due to haemorrhage and eclampsia. We assessed trends and determinants of three major complications and symptoms of preeclampsia, excessive bleeding, and convulsions, and related care-seeking practices.

**Methods:**

We analysed data from the 2001, 2010, and 2016 Bangladesh Maternal Mortality and Health Care Surveys, conducted as nationally representative cross-sectional surveys covering over 566,000 households. We conducted a secondary analysis using multivariable logistic regression to examine time trends and determinants of maternal complications and care-seeking practices.

**Results:**

In 2016, 49% of women reported complications during pregnancy, delivery, or postpartum. Further, 41% experienced at least one major complication – preeclampsia symptoms (37%), excessive bleeding (8%), and convulsions (6%). The likelihood of any major complication declined by 33% from 2001 to 2016. Risk factors for complications included women with primary or lower education, poor household wealth, multiple pregnancy, and four or more antenatal care (ANC) visits. Multiparous women (≥4 births) had higher odds of excessive bleeding and convulsions. Women with ≥4 ANC visits were more likely to experience excessive bleeding and convulsions. Care-seeking was higher among older women (aged ≥34 years), those with ≥4 ANC visits, and those who delivered at a facility, but lower among women with no education.

**Conclusions:**

Despite declining complication rates, care-seeking remains inadequate, especially among disadvantaged groups. Since women with no education exhibited the lowest care-seeking practices for pregnancy complications, promoting female education could play a crucial role in improving care-seeking behaviour. Additionally, improving quality ANC, facility readiness, and equity is critical to reducing preventable maternal deaths.

Globally, there is a remarkable reduction in maternal mortality from 339 deaths in 2000 to 197 deaths in 2023 per 100,000 live births [1], but the rate is still elevated, particularly in low- and middle-income countries (LMICs). Approximately 287,000 women died during pregnancy or in the postpartum period, with two major causes in 2020 – haemorrhage and eclampsia. 95% of these deaths were reported in LMICs. In 2023, around 87% (n = 225,000) of the estimated global maternal deaths were reported in Sub-Saharan Africa and Southern Asia, while most of the deaths were preventable [2].

Bangladesh has also experienced a huge reduction in the maternal mortality rate. According to the Bangladesh maternal mortality survey 2016, the maternal mortality rate dropped from 322 to 196 deaths per 100,000 live births from 2001 to 2016 [3]. Still, Bangladesh has a long way to go to achieve the Sustainable Development Goal (SDG), which targets decreasing the maternal mortality ratio (MMR) to <70 per 100,000 live births by 2030 [4,5]. In Bangladesh, approximately 3.3 million births occur annually, resulting in around 6500 maternal deaths [6], primarily due to haemorrhage (31%) and eclampsia (24%). Women aged 15–49 years experience pre-eclampsia symptoms, bleeding, and convulsion/fit during pregnancy, delivery or after delivery, which may lead to maternal death due to haemorrhage and eclampsia [7].

Maternal death as a result of haemorrhage refers to excessive bleeding from the reproductive tract during pregnancy, childbirth, or after delivery (within 42 days). Over half of antepartum haemorrhage (APH) cases are due to placenta previa or placental abruption. At the same time, a retained placenta primarily causes postpartum haemorrhage (PPH) [8,9]. APH and PPH are the major contributors to maternal mortality worldwide, particularly in South Asia [10,11]. Bangladesh affirmed its dedication to achieving the 2030 SDGs and declared a roadmap for ending preventable maternal mortality [11,12]. Although the MMR due to haemorrhage dropped at an average annual rate of 5% from 2001, this rate slowed significantly to just 2.1% after 2010. APH and PPH both cause one-third of maternal mortality in Bangladesh, with vast geographical and wealth inequalities [3,13]. Similar patterns are observed globally, where haemorrhage accounts for 27% of maternal deaths worldwide and 30% in the Southwestern Asia region [14]. According to the Bangladesh maternal mortality survey 2016, the cause-specific mortality ratios due to haemorrhage remained unchanged between 2010 and 2016, and it was 61 deaths per 100,000 live births [3]. The ratio remains very high and is one of the major barriers to achieving the SDG goal by 2030.

Another burden on the way to reducing maternal mortality is eclampsia, which accounts for 14% of maternal deaths globally. Of these, 99% of maternal deaths due to eclampsia occurred in developing countries, with most deaths occurring in Southern Asia (23%) and sub-Saharan Africa (61%) [15]. Despite the global reduction in maternal mortality, preeclampsia/eclampsia remains a leading cause of maternal death, with most deaths occurring in developing regions such as sub-Saharan Africa and Southern Asia [15]. According to the Bangladesh maternal mortality survey 2016, it is the second leading cause of maternal deaths in Bangladesh, which had a consistent position in both the Bangladesh Maternal Mortality and Health Care Surveys (BMMS) 2001 and 2010 rounds [3]. The report showed that the mortality ratio for eclampsia rose from 39 per 100,000 live births in BMMS 2010 to 46 per 100,000 live births.

Early identification of pregnancy-related complications can save maternal and newborn lives. Antenatal care (ANC) is a key policy for identifying pregnant women with complications. According to the World Health Organization (WHO), it is ideal for a pregnant woman to have at least four ANC visits during pregnancy. However, the Bangladesh Demographic Health Survey 2022 reported that only 41% of women who were pregnant received at least four ANC visits, which is very low. Additionally, numerous issues with care-seeking practices exist [16]. Many people do not know where to take pregnant women with complications for treatment. Furthermore, many mothers are either unaware of or ignore the signs of pregnancy-related complications. When their condition worsens, they are often taken to the nearest hospital, where most facilities are not equipped to handle such complex cases. Therefore, they are moved to another hospital, and over time, their situation deteriorates. One in five of them dies on the way to the hospital [17].

To effectively prevent and manage major causes of maternal death, such as APH, PPH, and eclampsia, the Bangladesh government has to realign its priorities as well as review the current plan of action designs. This demands a clear understanding of current challenges in care-seeking behaviour, awareness, access to services, and the quality of care that is provided to women [17]. By reducing deaths related to haemorrhage and eclampsia, which constitute 55% of maternal deaths in Bangladesh, significant declines in maternal mortality can be achieved. Therefore, early detection of complications like symptoms of pre-eclampsia, bleeding, and convulsions/fits, which tend to be the primary causes of haemorrhage and eclampsia, is crucial. To date, no study in Bangladesh has focused on these three complications during pregnancy, delivery, and after delivery, and their associated factors. Furthermore, there is no evidence on the care-seeking practices related to these complications and their associated factors. Therefore, we aimed to present levels, trends, and determinants of the three major maternal complications (excessive bleeding, symptoms of preeclampsia, and convulsions/fits) and their care-seeking practices.

## METHODS

We conducted a secondary analysis of data from the 2001, 2010, and 2016 BMMS, which were nationally representative cross-sectional surveys covering over 566,000 households. The National Institute of Population Research and Training conducted the three surveys with technical assistance from the International Centre for Diarrhoeal Disease Research, Bangladesh (icddr,b), the Monitoring and Evaluation to Assess and Use Results program, and several other organisations. The United States Agency for International Development funded the surveys, along with the Government of the People’s Republic of Bangladesh and other organisations [3].

### Sample selection

We included women of reproductive age (aged 13–49 years) who had their last live birth within three years prior to the survey. The selection criteria were met by 36,277 (in 2001), 17,151 (in 2010), and 27,133 (in 2016) women. In total, we included 80,561 reproductive-aged women.

### Outcome variables

The major complications such as symptoms of preeclampsia, excessive bleeding, and convulsions/fits along with any complication experienced during: pregnancy (the period from conception until the onset of labour where pregnancy ends when labour begins, not at birth), delivery (the period from onset of true labour until expulsion of the placenta), or the postpartum period (the period beginning immediately after the delivery of the placenta and continuing until the reproductive organs return to the non-pregnant state; the official puerperium lasts 42 days). We considered these as the primary outcome variables related to pregnancy complications. Symptoms of preeclampsia include severe headache, blurred vision, high blood pressure, or oedema of the face/feet/body. Excessive bleeding is referred to as bleeding from the genital tract during pregnancy (antepartum), birth (intrapartum), or within 42 days after birth (postpartum). A convulsion is a medical condition where the body’s muscles contract and relax rapidly and repeatedly, resulting in uncontrolled shaking. We included only women who reported experiencing convulsions/fits, irrespective of having or not having the symptoms of preeclampsia. In addition, we considered care-seeking behaviour for these complications as a second outcome of interest that was defined as a mother receiving the care from any provider or a medically trained provider.

### Independent variables

We considered maternal education, wealth quintile, administrative division, place of residence, birth order, religion, region, ANC visits, place of delivery, delivery mode, and mother’s age as the covariates based on the literature review for this study. Mother’s age, education, birth order, and number of ANC visits were continuous variables that were categorised into groups. We calculated the wealth index using principal component analysis and divided it into five categories from lowest to highest [18,19]. To address the geographical variation, we incorporated the division in the analysis. In 2001 and 2010, Bangladesh had six administrative divisions, with Mymensingh part of Dhaka and Rangpur in Rajshahi. But in 2016, there were eight administrative divisions, including Mymensingh and Rangpur. To maintain consistency across survey rounds, we recoded division in the 2016 BMMS rounds into six divisions similar to the BMMS 2001 and 2010 rounds. Religion was recoded as Muslim and others. We based the categorisation of all covariates on existing literature.

### Analysis plan

We performed the univariate analysis to examine the distribution of women’s background characteristics. To explore the patterns of maternal complications and care-seeking practices across the stages of pregnancy, delivery, and the postpartum period, we conducted a bivariate analysis over three BMMS rounds. Also, the percentage of care-seeking behaviour from any provider and a medically trained provider was shown using a bar chart.

To assess whether the time period affected pregnancy complications and care-seeking behaviour, we conducted multivariable logistic regression, and we reported adjusted odds ratios (aORs) with 95% confidence intervals (CIs). Additionally, to identify determinants of complications and care-seeking behaviour, we applied multivariable logistic regression to estimate aORs with 95% CIs, using the BMMS 2016 round. Statistical significance was considered when the *P*-value <0.05 or the CI of the aOR did not include ‘1’. We used a forest plot to illustrate the aOR along with a CI. We conducted data analysis using Stata, version 18.0 (StataCorp LLC., College Station, Texas, USA).

## RESULTS

The median age of the women was 25 years in the 2001 and 2010 surveys and 24 years in the 2016 survey. The majority of the women (41%) had secondary incomplete in 2016, compared with 6% in 2010 and 4% in 2001. Further, 39% of women aged 13–49 years had first-order births in 2016, compared with 34% in 2010 and 29% in 2001. The majority of women were Muslim. A substantial increase in the percentage of women receiving at least four ANC visits before childbirth occurred between 2001 and 2016, increasing from 12% to 37% (**Table 1**). Facility delivery has increased sharply from 9% in 2001 to 48% in 2016. Remarkably, the share of deliveries in private facilities had shown a 10-fold increase (from 3% to 30%) from 2001 to 2016, whereas deliveries in public facilities increased by nearly 3-fold (from 6% to 14%).

**Table 1:** Background characteristics of women of reproductive age (13–49 years) who had live births in the three years preceding the survey*

	BMMS 2016	BMMS 2010	BMMS 2001
**Mother’s age in years**			
13–23	12,816 (47.2)	7963 (46.4)	17,252 (47.6)
24–33	12,280 (45.3)	7587 (44.2)	14,796 (40.8)
34–49	2037 (7.5)	1601 (9.3)	4230 (11.7)
Mdn (IQR)	24 (8)	25 (8)	25 (10)
**Mother’s education**			
No education	2282 (8.4)	3924 (22.9)	16,008 (44.1)
Primary incomplete	4190 (15.4)	2739 (16.0)	6743 (18.6)
Primary complete	4108 (15.1)	8717 (50.8)	10,999 (30.3)
Secondary incomplete	11,081 (40.8)	1024 (6.0)	1479 (4.1)
Secondary complete/higher	5471 (20.2)	747 (4.4)	1033 (2.9)
**Wealth index**			
Lowest	5457 (20.1)	3833 (22.3)	9760 (26.9)
Second	5517 (20.3)	2158 (12.6)	4117 (11.3)
Middle	5353 (19.7)	3545 (20.7)	6703 (18.5)
Fourth	5589 (20.6)	3162 (18.4)	7806 (21.5)
Highest	5216 (19.2)	4454 (26.0)	7891 (21.8)
**Division**			
Barishal	1532 (5.6)	1004 (5.9)	2457 (6.8)
Chittagong	6245 (23.0)	3896 (22.7)	7544 (20.8)
Dhaka	9110 (33.6)	5671 (33.1)	12,343 (34.0)
Khulna	2557 (9.4)	1622 (9.5)	3530 (9.7)
Rajshahi	5705 (21.0)	3628 (21.2)	7811 (21.5)
Sylhet	1985 (7.3)	1330 (7.8)	2594 (7.1)
**Birth order**			
1	10,437 (38.5)	5860 (34.2)	10,375 (28.6)
2	8841 (32.6)	5049 (29.4)	8949 (24.7)
3	4631 (17.1)	2944 (17.2)	6231 (17.2)
≥4	3225 (11.9)	3298 (19.2)	10,723 (29.6)
**Religion**			
Muslim	25,139 (92.6)	15,697 (91.5)	33,405 (92.1)
Other	1994 (7.4)	1454 (8.5)	2873 (7.9)
**Region**			
Rural	19,964 (73.6)	13,166 (76.8)	29,961 (82.6)
Urban	7169 (26.4)	3985 (23.2)	6317 (17.4)
**ANC visits**			
No visit/NA*	4518 (16.7)	4948 (28.8)	18,765 (51.7)
1	3666 (13.5)	2976 (17.4)	5205 (14.3)
2	4586 (16.9)	2737 (16.0)	4519 (12.5)
3	4265 (15.7)	2468 (14.4)	3445 (9.5)
≥4	10,097 (37.2)	4022 (23.4)	4344 (12.0)
**Place of delivery**			
Home	14,130 (52.1)	12,995 (75.8)	33,035 (91.1)
Public	3876 (14.3)	1748 (10.2)	2015 (5.6)
NGO	906 (3.3)	358 (2.1)	144 (0.4)
Private	8222 (30.3)	2050 (12.0)	1081 (3.0)
**Mode of delivery**			
Single	26,902 (99.1)	17,018 (99.2)	35,935 (99.1)
Twin	231 (0.9)	133 (0.8)	343 (0.9)

ANC – antenatal care, BMMS – Bangladesh Maternal Mortality Survey, IQR – interquartile range, Mdn – median, NA – not available, NGO – non-government organisation *Presented as n (%) unless specified otherwise.

Nearly half of the women (52% in 2001, 53% in 2010, and 49% in 2016) developed complications during any stage of their pregnancy in the three years preceding the survey. Among women who experienced any of the three major complications, 41% reported at least one. Most complications occurred during the antepartum period (33%), followed by the intrapartum period (17%) and the postpartum period (20%). The prevalence of the three major complications was slightly higher in earlier rounds, at 43% in 2010 and 46% in 2001. A similar pattern of complications across the pregnancy stages, including antepartum, intrapartum, and postpartum, was observed in both 2001 and 2010.

In 2016, 37% of women reported experiencing symptoms of preeclampsia at any stage of their pregnancy, during or after delivery. Nearly one-third (32%) of these women suffered from preeclampsia symptoms during the antepartum period, while 14% experienced symptoms during the intrapartum and postpartum periods, respectively. Additionally, 8% of pregnancies were complicated by severe excessive bleeding at any stage of ante, intra, or postpartum periods, with 6% of women experiencing excessive bleeding, especially during the postpartum period. Convulsions or fits were reported by 2–3% of women during ante, intra, or postpartum periods, totalling 6% overall (**Table 2**).

**Table 2:** Maternal complications of women of reproductive age (13–49 years) during the stage of pregnancy, delivery, and after delivery in 2001, 2010, and 2016, %

Complications	During pregnancy	During delivery	After delivery	Any stage
BMMS 2016 (n = 27,133)				
*Any complication (all complications)*	37.4	25.7	20.7	49.3
*Any of major three complications*	32.9	16.7	19.7	41.3
*Symptoms of preeclampsia*	31.5	14.4	14	36.5
*Severe/heavy/excessive bleeding*	1.3	2.2	5.8	7.9
*Convulsion/fits*	2.1	1.5	3.3	6.2
BMMS 2010 (n = 17,151)				
*Any complication (all complications)*	40.1	28	19.9	52.9
*Any of major three complications*	36.1	16.8	18.2	43
*Symptoms of preeclampsia*	34.5	13.6	12	37.8
*Severe/heavy/excessive bleeding*	1.5	3.3	6.5	9.4
*Convulsion/fits*	2.3	1.7	3	6.4
BMMS 2001 (n = 36,277)				
*Any complication (all complications)*	45.5	23.4	23.4	52.4
*Any of major three complications*	39.7	15.3	18	46.6
*Symptoms of preeclampsia*	38.6	10.9	7.8	40.8
*Severe/heavy/excessive bleeding*	1.5	4.5	10.3	13.3
*Convulsion/fits*	1.3	1.6	3.1	5.3

BMMS – Bangladesh Maternal Mortality Survey

Furthermore, 62% of women who experienced any of the three major complications sought healthcare, while only 41% reported receiving care from medically trained providers in 2016. Less than half of the women with symptoms of preeclampsia (40%) and excessive bleeding (46%) received healthcare from a medically trained provider, whereas it was 52% for convulsions or fits (**Table 3**).

**Table 3:** Care seeking practices of women of reproductive age (13–49 years) for maternal complications during any stage of pregnancy, delivery, and after delivery in 2016, %

Complication	Care sought from any provider	Care sought from medically trained provider
Any of major three complications	62.0	41.0
Symptoms of preeclampsia	60.0	40.0
Severe/heavy/excessive bleeding	78.0	46.0
Convulsion/fits	78.0	52.0

The probability of developing any of three major complications decreased by 20% in 2010 (aOR = 0.8; 95% CI = 0.75–0.81) and by 30% in 2016 (aOR = 0.7; 95% CI = 0.64–0.70) compared with 2001. The odds of having symptoms of preeclampsia also decreased in 2010 (aOR = 0.8; 95% CI = 0.77–0.83) and 2016 (aOR = 0.7; 95% CI = 0.67–0.73) compared with 2001. The likelihood of experiencing bleeding decreased by 30% in 2010 (aOR = 0.7; 95% CI = 0.61–0.69) and was halved in 2016 (aOR = 0.5; 95% CI = 0.51–0.58) compared with 2001. However, the odds of convulsions increased by 10% in 2010 (aOR = 1.1; 95% CI = 1.05–1.24) compared with 2001.

**Figure 1:**
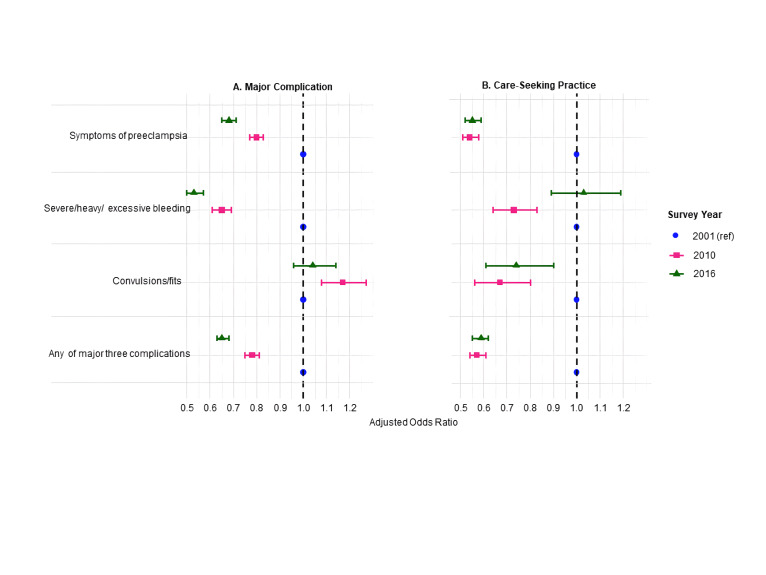
Adjusted odds ratio of maternal major three complications. **Panel A.** Among reproductive-age women (13–49 years) during any stage of pregnancy, delivery, and after delivery and their care-seeking practice. **Panel B.** In 2010 and 2016 compared to 2001.

The probability of seeking healthcare for any of these complications decreased by 40% both in 2010 (aOR = 0.6; 95% CI = 0.54–0.61) and 2016 (aOR = 0.6; 95% CI = 0.52–0.60) compared with 2001. Women with symptoms of preeclampsia were 50% less likely to seek healthcare in both 2010 (aOR = 0.5; 95% CI = 0.51–0.58) and 2016 (aOR = 0.5; 95% CI = 0.50–0.57) compared with 2001. The likelihood of seeking care for convulsions decreased by 30% both in 2010 (aOR = 0.7; 95% CI = 0.57–0.81) and 2016 (aOR = 0.7; 95% CI = 0.55–0.84) compared with 2001 (**Figure 1**).

**Figure 2:**
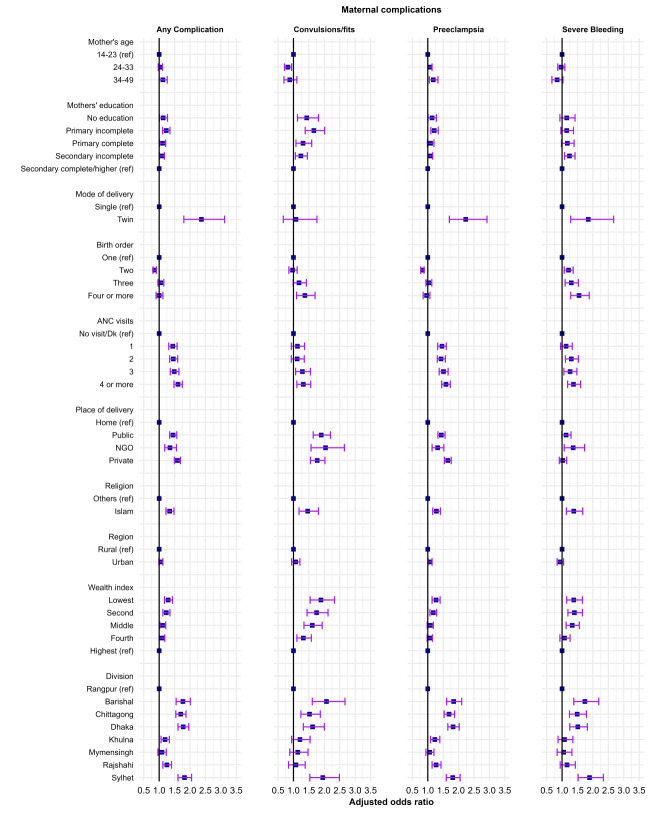
Risk factors of maternal major three complications among reproductive age (13–49 years) during any stage of pregnancy, delivery, and after delivery using the 2016 round of BMMS. BMMS – Bangladesh Maternal Mortality and Health Care Survey.

Mother’s education had a significant association with having any one of the three complications (symptoms of preeclampsia, excessive bleeding, and convulsions) during pregnancy or during delivery or after delivery; as mothers who had not completed primary education had 23% more likely (aOR = 1.23; 95% CI = 1.11–1.35) to suffer from any one of three complications and 66% more likely of having convulsions (aOR = 1.66; 95% CI = 1.38–2.01) compared to mothers who had secondary complete or higher education. Simultaneously, the odds of having any one of three complications among mothers increased for women who belonged to the lower wealth quintile. Mothers in the lowest wealth quintile (aOR = 1.29; 95% CI = 1.17–1.43) were more likely to have any one of three complications compared with those from the highest wealth quintile. The patterns are similar for symptoms of preeclampsia (aOR = 1.27; 95% CI = 1.14–1.40) and excessive bleeding (aOR = 1.38; 95% CI = 1.15–1.66) for the lowest wealth categories, however, those women were more likely to have convulsions (aOR = 1.89; 95% CI = 1.54–2.33) in the highest wealth quintile group. Multiparous women (≥4) had a greater likelihood of having excessive bleeding (aOR = 1.55; 95% CI = 1.28–1.88) and convulsions (aOR = 1.37; 95% CI = 1.1–1.7). ANC visits were associated with maternal major complications, as mothers who reported receiving ≥4 ANC visits had 59% more likelihood of having symptoms of preeclampsia (aOR = 1.59; 95% CI = 1.45–1.73), excessive bleeding (aOR = 1.37; 95% CI = 1.18–1.6), and convulsions (aOR = 1.32; 95% CI = 1.11–1.56). Mothers delivered at a facility (aOR = 1.65; 95% CI = 1.54–1.76) and delivered twins or more (aOR = 1.65; 95% CI = 1.54–1.76) were more likely to suffer from excessive bleeding (**Figure 2**).

**Figure 3:**
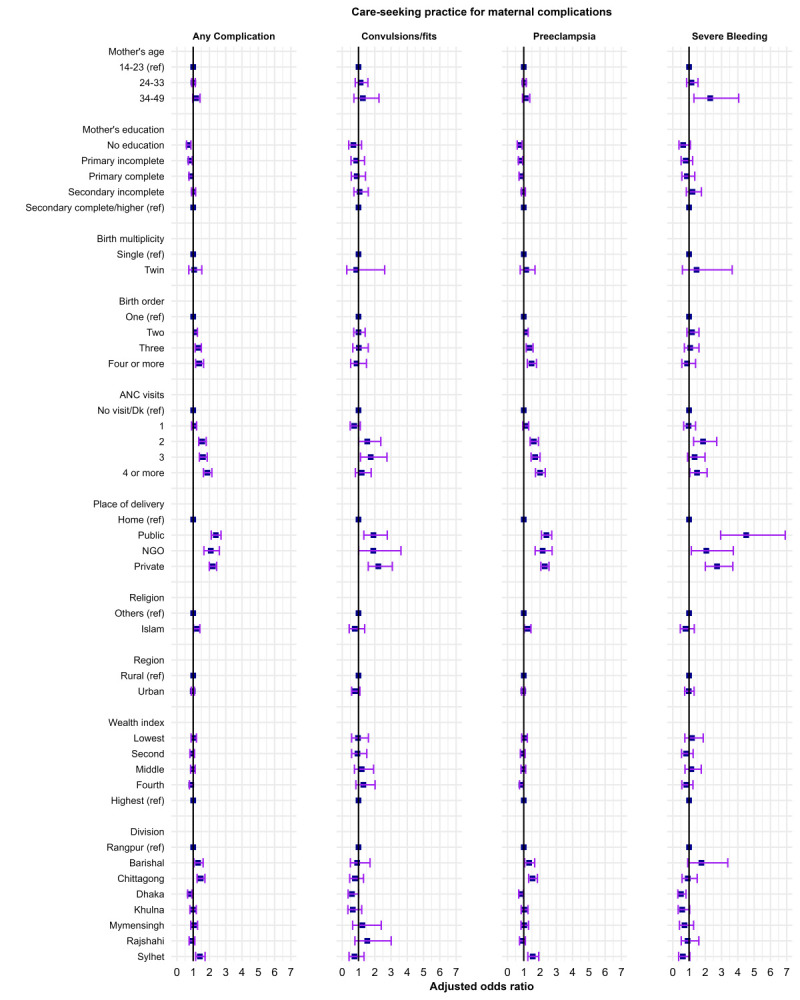
Determinants of care-seeking practice for maternal major three complications using the 2016 round of BMMS. BMMS – Bangladesh Maternal Mortality and Health Care Survey.

Care-seeking practice for older women aged ≥34 years with excessive bleeding was higher (aOR = 2.3; 95% CI = 1.3–4.05) compared to younger women aged ≤23 years. The odds of seeking care for any of the three major complications are lower (aOR = 0.7; 95% CI = 0.6–0.9) among the women with no education. The odds of seeking treatment for symptoms of preeclampsia were lower in different educational attainment (*e.g.* primary complete, incomplete, or no education) compared to mothers who have secondary complete or higher education. Mothers with a higher parity were more likely to seek treatment for preeclampsia symptoms, with the likelihood being 1.5 times higher for women with a birth order of four or higher compared to those with their first child. The likelihood of seeking treatment for any of the three complications increased with the number of ANC visits. Mothers who reported receiving at least four ANC visits had an 87% higher likelihood compared to those with fewer than four ANC visits (aOR = 1.87; 95% CI = 1.63–2.15). Mothers who had facility delivery had higher odds of seeking treatment for any of the three complications in contrast to the mothers who had home delivery. The odds were highest across women who had delivery in a public facility for any of the three complications (aOR = 2.39; 95% CI = 2.11–2.71) which varies for those having symptoms of preeclampsia (aOR = 2.39; 95% CI = 2.09–2.72), excessive bleeding (aOR = 4.51; 95% CI = 2.94–6.91), and convulsions/fit (aOR = 1.92; 95% CI = 1.33–2.77) (**Figure 3**).

## DISCUSSION

There has been notable improvement in maternal health outcomes in Bangladesh over the past few years [20]. The MMR significantly decreased, with an average annual rate of decrease of 5.1%. But this reduction plateaued from 2010 to 2016, leaving gaps in prioritisation between maternal health interventions and health system strategies. This situation reflects the importance of reviewing and improving the current strategies to better promote maternal health [21]. To date, this is the first nationally representative study in Bangladesh to systematically explore the three clinically recognisable maternal complications, namely symptoms of preeclampsia, excessive bleeding, and convulsions, across all stages of pregnancy through postpartum over fifteen years. While earlier studies only focused on primary causes of maternal mortality, this study explores the prevalence of complications with care-seeking behaviours at the population level. By disaggregating data by maternal stage and controlling for socioeconomic, demographic, and service-related factors, we address the key evidence gap and offer actionable insights to improve maternal care.

We found that around half of women reported complications during pregnancy, delivery, or postpartum, with a significant proportion experiencing at least one of the critical conditions of preeclampsia, haemorrhage, or convulsions. Although the adjusted likelihood of these complications had reduced over time, accessing care from medically qualified providers remained minimal. In 2016, less than half of the women with serious complications received care from skilled providers, indicating that progress in maternal mortality indicators did not close gaps in identification and response.

The trend finds that while raw care-seeking behaviour rates have improved, the adjusted probabilities of care-seeking behaviour have declined. While unadjusted data show an increase in service utilisation from the medically trained provider for any of the three major complications from 28% in 2001 to 41% in 2016, adjusted models reveal a decrease in the likelihood of care-seeking, suggesting that improvements have not reached the most vulnerable groups (Table S1 in the **Online Supplementary Document**). A cohort study in rural Bangladesh found that although 61% of women with intrapartum complications received any care, fewer than 50% accessed skilled providers [22]. Wealth, literacy, and accessibility to facilities were significant components of access to skilled care. Similarly, in northwest Bangladesh, fewer than 30% of women with haemorrhage or obstructed labour received formal care, with only modest improvement over time [23]. Together, these findings confirm that while overall care-seeking is on the rise, inequities persist.

Our study shows that one in three pregnant women in Bangladesh had symptoms of preeclampsia, whereas one in eight experienced excessive bleeding. Haemorrhage and eclampsia are the most significant complications in Bangladesh. According to the causes of maternal mortality research in Bangladesh, haemorrhage causes 31% and eclampsia 24% of maternal deaths in recent years [3,24]. Notable gaps remain in birth preparedness and complication readiness in pregnant women and their families in Bangladesh [25]. It is reported that over 60% of maternal mortality in Bangladesh is caused by haemorrhage and occurs during home delivery with unskilled healthcare providers [17]. ANC visits give a major opportunity to recognise complicated pregnancies and provide knowledge to family members about potential danger signs [26]. Anaemia occurring in pregnancy is a common risk factor for maternal deaths related to APH and PPH. For this reason, measuring haemoglobin levels during ANC is essential for diagnosing anaemia [26]. In Bangladesh, quality antenatal care is not sufficient to identify high-risk pregnancies like maternal anaemia [16]. In addition, the health system’s readiness is also insufficient to determine pregnancy complications such as maternal anaemia, with only 19% of facilities providing antenatal care being ready to detect blood haemoglobin levels [27,28]. Only 50% of pregnant women were provided with danger signs related to pregnancy complications counselling during their antenatal care visits [16]. The results of other studies also support findings similar to those for preeclampsia in pregnancy [29].

We found that nearly 60% of pregnant women with symptoms of preeclampsia and 48% of those experiencing convulsions/fits did not seek care, highlighting a lack of recognition of the urgency for treatment to prevent serious complications. Despite the serious risk of convulsions, three in five women who gave live birth did not recognise the convulsions [3]. Furthermore, over 40% of women with preeclampsia and 15% with convulsions did not receive treatment, mostly because they overlooked the severity of these complications [3]. These findings are nearly consistent with our results, highlighting the need to improve awareness and timely care-seeking for maternal complications. Although 80% of women had at least one ANC visit from a medically trained provider, only about 40% received information about the danger signs of pregnancy complications, highlighting a significant missed opportunity [30]. A urine protein test, an important diagnostic tool for eclampsia, was not performed on over one-fourth of the women who attended antenatal care visits [31]. According to a study finding, 93% of deaths caused by eclampsia happened at any health facility or during transportation. This shows that treatment was sought only when the illness became very serious [31]. This suggests that they would have sought institutional care if they had been able to comprehend the severity. Therefore, the first approach in preventing eclampsia mortality during ANC could be counselling on the symptoms of eclampsia and its potentially fatal risk [31]. One third of private (36%) and around 84% of public health facilities providing normal delivery services lacked anticonvulsants, indicating a deficiency in readiness to combat eclampsia cases [27]. In the 2022 survey, it was also found that only 17% of the government union-level facilities had injectable magnesium sulphate [27]. Research in sub-Saharan Africa reveals similar complications and care-seeking dynamics. For example, a study on adolescent pregnancies shows that eclampsia remains a major contributor to maternal mortality and is associated with delayed ANC and poor access to effective medical interventions [32]. Several studies in Uganda and Senegal demonstrated how geographic and financial barriers, cultural norms, and perceived quality of care confine facility deliveries and timely responses to complications [33,34]. To prevent maternal deaths caused by haemorrhage and eclampsia, existing policies and strategies need to be reviewed and improved to overcome current challenges. This study showed that leading maternal complications are closely associated with factors like the mother’s education, family income, number of ANC visits, delivery locations, and delivery mode. It also showed that care-seeking behaviour among women with complications was affected by the mother’s age, education, number of children, ANC visits, and the delivery place [17].

The place of delivery also shapes outcomes. While our findings show that women who deliver in facilities were more likely to report and receive treatment for complications, other studies indicate that many complications actually begin at home and are only brought to facilities when they become severe, sometimes leading to fatalities during transit or shortly after arrival [35]. This highlights the critical importance of seeking care immediately when complications arise at home. This aligns with the three delays model, which was applied in Uganda, where scarcity of decision-making power, lack of vehicles, and insufficient facility readiness contributed to maternal deaths [36]. Our findings also show that the likelihood of experiencing any of the three leading complications: excessive bleeding, symptoms of pre-eclampsia, and convulsions/fits, is increased 2-fold for women who delivered twins or more compared to those with singleton pregnancies. A study conducted in 23 LMICs identified that twin pregnancies remarkably increased the risk of severe maternal outcomes, including haemorrhage and eclampsia [37].

Socioeconomic disparities significantly influence both the occurrence of maternal complications and the likelihood of seeking care [38]. The poorest women had higher rates of complications and were less likely to receive quality antenatal care or deliver at a facility [16,23]. For instance, only 8% of women from the poorest wealth group received quality ANC compared to 39% from the richest group, and facility delivery rates ranged from 42% in the lowest quintile to 87% in the highest [16]. Our study also indicates that mothers from the lowest economic groups were more likely to have any of these complications, such as symptoms of preeclampsia, excessive bleeding, and convulsion/fits, and less likely to seek care compared to those from the highest economic groups (Tables S2 and S3 in the **Online Supplementary Document**). Global evidence finds that women with a lower educational level are more prone to suffer from complications such as preeclampsia, bleeding, and convulsions, and are also less likely to seek timely care [39], which also supports our findings. Education affects a woman’s ability to identify danger signs, navigate the health system, and make informed decisions about care-seeking help. Similarly, supporting the global evidence, household wealth showed a strong correlation with maternal health outcomes [40]. These disparities are responsible for unequal health outcomes. However, once complications become severe, care-seeking behaviour may become more consistent across wealth groups, as families take urgent steps such as borrowing or selling assets to save the mother’s life [40–42]. Age was also a determinant. Elderly women were both more likely to report complications, particularly symptoms of preeclampsia, and were also more likely to access care, possibly due to increased awareness or previous experience [43]. These inequalities must be tackled with targeted education, financial protection schemes, and increased outreach to poor and low-literacy populations to reduce preventable maternal morbidity and mortality.

To reduce the excessive maternal complications, there are significant evidence-based interventions that can be executed in addition to the integrated care pathway. The ANC quality must be enhanced, including measurement of blood pressure on a regular basis, proteinuria screening through urine tests, and anaemia screening to identify the early symptoms of preeclampsia and risk of haemorrhage. Community health workers play a vital role in raising awareness of danger signs and promoting timely care-seeking, especially among women in remote or hard-to-reach areas. Emergency obstetric care services must be made more accessible and better equipped, especially with basic medicines such as magnesium sulphate for eclampsia and uterotonic drugs to control bleeding. Skilled birth attendants and facility staff should also be equipped to detect early complications and respond instantly. In addition, establishing efficient referral systems and community-based transportation support can minimise delays in receiving care. Schemes of financial support or conditional cash transfers can help overcome financial barriers to accessing skilled care. All the above interventions together can make a significant difference in early detection, timely treatment, and ultimately in minimising maternal complications and mortalities.

### Strengths and limitations

There are multiple significant strengths in this study. First, we used data collected across three nationally representative rounds of the BMMS, allowing us to conduct rigorous trend analysis over 15 years. The high sample size and systematic method of data collection provide the generalisability and validity of the findings. Second, the study identifies three principal maternal complications, namely excessive bleeding, symptoms of preeclampsia, and convulsions/fits, which are also the leading causes of maternal deaths in Bangladesh and worldwide. Disaggregating complications by pregnancy stage (antepartum, intrapartum, and postpartum) enabled us to develop a differentiated understanding of when and how these complications arise. Further, we explored care-seeking behaviours and their determinants, giving substantial insights into the enablers and challenges towards the utilisation of maternal healthcare. Although we utilised data collected a decade ago, maternal mortality estimates in Bangladesh are generated exclusively through the BMMS, and no comparable national survey has been conducted in the intervening years. Therefore, our findings provide critical evidence to inform policymakers and underscore the need for more regular implementation of such national surveys. This constitutes an important strength of the study.

However, there are some shortfalls. The data are subject to recall and self-reported symptoms, which are susceptible to misclassification and recall bias. Sometimes mothers may not accurately recall what constitutes heavy bleeding, or they may not have understood the term ‘heavy bleeding,’ as these were self-reported statements. Additionally, the surveys did not have clinical validation for complications that may result in under- or over-reporting. Nevertheless, it may also be the case that the symptoms were noted by women through prior knowledge reported by family members (*e.g.* mothers or in-laws) or ANC visits. Finally, while the analysis establishes a correlation between various determinants and maternal complications or care-seeking behaviour, it cannot establish causality due to its cross-sectional design.

## CONCLUSIONS

This study provides novel and robust findings on the major maternal complications in Bangladesh and the poor care-seeking response. Though the complication rate has marginally decreased, nearly half of the women still experience severe maternal symptoms. Though unadjusted care-seeking behaviour appears to be increasing, adjusted models demonstrate a falling probability, a sign of disparity. There must be specific interventions to improve the quality of the ANC, enable early detection and management of complications, invest in facility readiness, and address system-level issues such as cost, transport, and cultural perceptions. Interventions that were found in other settings, such as emergency transport, community insurance, and a bundle of interventions for haemorrhage, must be piloted and scaled up in Bangladesh. Enhancing quality and equity of maternal care is imperative to achieve the SDG to decrease maternal mortality to below seventy per hundred thousand by 2030.

## Additional material


Online Supplementary Document

## Data Availability

**Data availability:** The DHS Program and the MEASURE Evaluation provide public access to the data through their websites.
