# Supplementary material for: Navigating maternal health challenges in Bangladesh: an analysis of pregnancy complications and care-seeking behaviours using nationally representative surveys
Source: J Glob Health. 2026 Aug 7;16:04261. doi: 10.7189/jogh.16.04261 (PMC13449554; doi:10.7189/jogh.16.04261)
Supplement: Supplementary file 1 [file jogh-16-04261-s001.pdf]

Supplement to: Siddique AB, Saha A, Haider MM, Hossain AT, Ashiquzzaman, Modasser RB, Lucky NZ, Kana AN, Mehjabin M, Ameen S, Ahmed A, Arifeen SE, Rahman AE. Navigating maternal health challenges in Bangladesh: an analysis of pregnancy complications and care-seeking behaviours using nationally representative surveys. J Glob Health. 2026;16:04261.

Table S1: Care seeking practice for top three major maternal complications during any stage of pregnancy, delivery, or after delivery

Complication	Number of women suffering	Care sought from any provider (%)	Care sought from medical trained provider (%)
BMMS 2016			
Any of major three complications	11211	61.9	40.5
Symptoms of preeclampsia	9894	59.5	39.6
Severe/heavy/excessive bleeding	2147	78.0	46.3
Convulsion/fits	1685	78.3	52.3
BMMS 2010			
Any of major three complications	7381	51.7	33.9
Symptoms of preeclampsia	6483	49.1	32.9
Severe/heavy/excessive bleeding	1606	63.9	39.7
Convulsion/fits	1090	67.9	42.1
BMMS 2001			
Any of major three complications	16918	54.6	28.1
Symptoms of preeclampsia	14806	53.3	27.7
Severe/heavy/excessive bleeding	4822	60.1	28.1
Convulsion/fits	1914	71.0	35.3

Table S2: Risk factors of maternal major three complications using the 2016th round of BMMS

Exposures	Any three complication		Symptoms of preeclampsia		Severe/heavy/ excessive bleeding		Convulsions/fits	
	%	AOR (95% CI)	%	AOR (95% CI)	%	AOR (95% CI)	%	AOR (95% CI)
**Mother's age**								
14-23	40.9	*Ref*	35.8	*Ref*	7.5	*Ref*	6.4	*Ref*
24-33	41.4	1.04 (0.97, 1.11)	36.6	1.07 (1.00, 1.14)	8.4	0.97 (0.86, 1.09)	5.8	**0.82 (0.71, 0.94)**
34-49	44	**1.12 (1.00, 1.26)**	39.5	**1.18 (1.05, 1.33)**	8.0	0.84 (0.67, 1.03)	7.2	0.88 (0.69, 1.11)
**Mothers' Education**								
No education	39.7	**1.13 (1.01, 1.27)**	35.0	**1.14 (1.01, 1.28)**	8.4	1.15 (0.93, 1.42)	6.8	**1.43 (1.13, 1.81)**
Primary incomplete	42.3	**1.23 (1.11, 1.35)**	37.1	**1.21 (1.1, 1.34)**	8.3	1.15 (0.96, 1.37)	7.9	**1.66 (1.38, 2.01)**
Primary complete	40.4	**1.11 (1.01, 1.21)**	35.2	1.09 (0.99, 1.2)	8.3	1.17 (0.98, 1.39)	6.4	**1.31 (1.08, 1.59)**

Exposures	Any three complication		Symptoms of preeclampsia		Severe/heavy/ excessive bleeding		Convulsions/fits	
	%	AOR (95% CI)	%	AOR (95% CI)	%	AOR (95% CI)	%	AOR (95% CI)
Secondary incomplete	41.1	**1.08 (1.01, 1.17)**	36.2	**1.08 (1.00, 1.16)**	8.3	**1.24 (1.08, 1.42)**	6.0	**1.24 (1.06, 1.45)**
Secondary complete/higher	42.3	*Ref*	38.1	*Ref*	6.2	*Ref*	4.9	*Ref*
**Wealth index**								
Lowest	39.4	**1.29 (1.17, 1.43)**	34.4	**1.27 (1.14, 1.40)**	8.6	**1.38 (1.15, 1.66)**	6.9	**1.89 (1.54, 2.33)**
Second	40.2	**1.23 (1.12, 1.35)**	34.9	**1.18 (1.07, 1.29)**	8.6	**1.4 (1.19, 1.66)**	6.7	**1.75 (1.44, 2.12)**
Middle	40.2	**1.11 (1.02, 1.21)**	35.2	1.08 (0.98, 1.18)	8.4	**1.33 (1.13, 1.56)**	6.4	**1.61 (1.34, 1.93)**
Fourth	42.6	**1.09 (1.00, 1.18)**	37.7	1.06 (0.98, 1.15)	7.2	1.08 (0.93, 1.26)	5.9	**1.32 (1.11, 1.58)**
Highest	44.3	*Ref*	40.2	*Ref*	6.8	*Ref*	5.0	*Ref*
**Division**								
Rangpur	33.2	*Ref*	28.4	*Ref*	6.3	*Ref*	4.8	*Ref*
Barishal	44.2	**1.77 (1.55, 2.01)**	39.7	**1.84 (1.61, 2.10)**	10.1	**1.74 (1.38, 2.19)**	8.6	**2.07 (1.61, 2.67)**
Chittagong	43.8	**1.7 (1.54, 1.87)**	38.4	**1.69 (1.53, 1.87)**	8.5	**1.49 (1.24, 1.79)**	6.2	**1.52 (1.24, 1.87)**
Dhaka	46.3	**1.78 (1.61, 1.96)**	41.8	**1.82 (1.65, 2.02)**	8.1	**1.51 (1.25, 1.82)**	6.7	**1.62 (1.32, 2)**
Khulna	37.6	**1.19 (1.06, 1.33)**	33.5	**1.23 (1.09, 1.39)**	6.5	1.08 (0.87, 1.35)	5.6	1.21 (0.94, 1.54)
Mymensingh	33.4	1.09 (0.96, 1.23)	28.0	1.06 (0.94, 1.2)	6.5	1.05 (0.84, 1.32)	5.2	1.14 (0.88, 1.47)
Rajshahi	37.9	**1.25 (1.12, 1.40)**	30.9	**1.27 (1.14, 1.43)**	6.7	1.16 (0.94, 1.43)	4.9	1.08 (0.84, 1.38)
Sylhet	44.4	**1.82 (1.61, 2.05)**	39	**1.81 (1.60, 2.05)**	10.7	**1.89 (1.52, 2.34)**	8.1	**1.95 (1.53, 2.49)**
**Birth order**								
One	42.7	*Ref*		*Ref*		*Ref*		*Ref*
Two	38.3	0.85 (0.80, 0.91)	33.4	0.83 (0.77, 0.88)	8.0	**1.21 (1.07, 1.36)**	5.5	0.97 (0.85, 1.12)
Three	43.4	1.05 (0.96, 1.15)	38.6	1.03 (0.94, 1.13)	8.7	**1.30 (1.10, 1.53)**	6.5	1.18 (0.99, 1.42)
Four or more	42.1	1.00 (0.90, 1.12)	36.8	0.96 (0.85, 1.07)	10.1	**1.55 (1.28, 1.88)**	7.6	**1.37 (1.1, 1.7)**
**Religion**								
Others	36.4	*Ref*	32.9	*Ref*	6.1	*Ref*	4.8	*Ref*
Muslim	41.7	**1.34 (1.22, 1.48)**	36.8	**1.28 (1.16, 1.41)**	8.1	**1.38 (1.14, 1.67)**	6.3	**1.46 (1.18, 1.81)**
**Region**								
Rural	40.4	*Ref*	35.4	*Ref*	8.1	*Ref*	6.2	*Ref*
Urban	43.9	1.06 (0.99, 1.12)	39.4	**1.07 (1.01, 1.14)**	7.3	0.94 (0.84, 1.05)	6.2	1.07 (0.94, 1.21)
**ANC visits**								
No visit/Dk	32.1	*Ref*	27.5	*Ref*	7.4	*Ref*	5.5	*Ref*
1	41.2	**1.44 (1.31, 1.58)**	36.5	**1.46 (1.32, 1.60)**	7.8	1.13 (0.95, 1.33)	6.1	1.13 (0.93, 1.36)
2	42.3	**1.46 (1.34, 1.60)**	37.0	**1.43 (1.31, 1.57)**	8.5	**1.30 (1.11, 1.53)**	6.0	1.12 (0.93, 1.35)

Exposures	Any three complication		Symptoms of preeclampsia		Severe/heavy/ excessive bleeding		Convulsions/fits	
	%	AOR (95% CI)	%	AOR (95% CI)	%	AOR (95% CI)	%	AOR (95% CI)
3	43.0	**1.49 (1.36, 1.64)**	38.5	**1.51 (1.37, 1.66)**	7.9	**1.26 (1.06, 1.48)**	6.8	**1.29 (1.07, 1.55)**
4 or more	44.3	**1.61 (1.48, 1.75)**	39.4	**1.59 (1.45, 1.73)**	7.9	**1.37 (1.18, 1.6)**	6.4	**1.32 (1.11, 1.56)**
**Place of delivery**								
Home	36.3	*Ref*	31.3	*Ref*	8.1	*Ref*	5.2	*Ref*
Public	45.3	**1.45 (1.34, 1.57)**	38.1	**1.44 (1.33, 1.56)**	8.2	1.13 (0.99, 1.29)	8.7	**1.9 (1.64, 2.2)**
NGO	45.1	**1.35 (1.18, 1.56)**	38.5	**1.32 (1.14, 1.52)**	5.3	**1.36 (1.07, 1.73)**	7.5	**2.04 (1.57, 2.65)**
Private	47.6	**1.59 (1.49, 1.69)**	37.6	**1.65 (1.54, 1.76)**	8.0	1.02 (0.91, 1.15)	6.3	**1.77 (1.55, 2.02)**
**Mode of delivery**								
Single	41.1	*Ref*	36.3	*Ref*	7.9	*Ref*	6.2	*Ref*
Twin	64.1	**2.37 (1.80, 3.12)**	57.7	**2.23 (1.70, 2.92)**	15.3	**1.85 (1.28, 2.67)**	8.1	1.08 (0.67, 1.76)

Table S3: Determinants of care seeking practice for maternal major 3 complications using the 2016th round of BMMS

Exposures	Any of major three complications		Symptoms of preeclampsia		Severe/heavy/ excessive bleeding		Convulsions/fits	
	%	AOR (95% CI)	%	AOR (95% CI)	%	AOR (95% CI)	%	AOR (95% CI)
**Mother's age**								
14-23	60.2	*Ref*	57.5	*Ref*	76.8	*Ref*	78.1	*Ref*
24-33	62.9	1.01 (0.91, 1.13)	60.9	1.03 (0.92, 1.15)	78.1	1.15 (0.85, 1.55)	78.7	1.13 (0.8, 1.58)
34-49	65.6	1.18 (0.97, 1.42)	63.1	1.12 (0.92, 1.37)	84.2	**2.3 (1.3, 4.05)**	77.4	1.27 (0.72, 2.26)
**Mother's Education**								
No education	52.7	**0.71 (0.59, 0.86)**	51.4	**0.73 (0.6, 0.89)**	67.2	0.64 (0.38, 1.08)	70.7	0.69 (0.4, 1.19)
Primary incomplete	54.6	**0.81 (0.7, 0.94)**	52.1	**0.79 (0.67, 0.93)**	70.3	0.79 (0.51, 1.22)	73.5	0.86 (0.54, 1.37)
Primary complete	57.1	**0.86 (0.74, 1)**	54.2	**0.84 (0.71, 0.98)**	74.0	0.87 (0.56, 1.35)	76.1	0.89 (0.56, 1.43)
Secondary incomplete	64.6	1.02 (0.9, 1.15)	61.8	0.97 (0.86, 1.1)	82.2	1.21 (0.83, 1.76)	81.3	1.07 (0.72, 1.6)
Secondary complete/higher	69.0	*Ref*	67.4	*Ref*	84.4	*Ref*	83.0	*Ref*
**Wealth index**								
Lowest	57.1	1.02 (0.87, 1.2)	54.5	1.03 (0.87, 1.22)	77.8	1.18 (0.74, 1.87)	73.7	0.97 (0.58, 1.61)
Second	58.1	0.93 (0.8, 1.08)	55.6	0.92 (0.79, 1.08)	72.1	0.82 (0.54, 1.25)	75.8	0.94 (0.58, 1.51)
Middle	62.7	0.97 (0.84, 1.12)	59.9	0.96 (0.83, 1.12)	81.9	1.14 (0.75, 1.75)	81.1	1.2 (0.75, 1.93)
Fourth	61.6	**0.88 (0.77, 1)**	58.9	**0.85 (0.74, 0.98)**	76.6	0.83 (0.56, 1.24)	81.8	1.3 (0.84, 2.02)
Highest	69.5	*Ref*	60.1	*Ref*	82.8	*Ref*	80.1	*Ref*
**Division**								
Rangpur	62.1	*Ref*	59.1	*Ref*	83.9	*Ref*	80.4	*Ref*

Exposures	Any of major three complications		Symptoms of preeclampsia		Severe/heavy/ excessive bleeding		Convulsions/fits	
	%	AOR (95% CI)	%	AOR (95% CI)	%	AOR (95% CI)	%	AOR (95% CI)
Barishal	63.1	1.3 (1.05, 1.61)	60.1	1.33 (1.06, 1.67)	87.6	1.76 (0.92, 3.38)	78.2	0.92 (0.5, 1.71)
Chittagong	68.1	1.46 (1.24, 1.72)	66.2	1.54 (1.3, 1.84)	81.6	0.92 (0.57, 1.5)	77.5	0.78 (0.46, 1.31)
Dhaka	56.9	0.78 (0.66, 0.92)	55.0	0.82 (0.69, 0.98)	71.3	0.5 (0.31, 0.81)	75.3	0.6 (0.36, 1.01)
Khulna	64.1	0.98 (0.8, 1.19)	61.7	1.03 (0.84, 1.26)	79.7	0.58 (0.33, 1.04)	79.1	0.65 (0.35, 1.2)
Mymensingh	58.3	1.04 (0.85, 1.27)	55.0	1.03 (0.83, 1.29)	73.3	0.72 (0.41, 1.28)	83.1	1.24 (0.64, 2.4)
Rajshahi	58.3	0.91 (0.75, 1.1)	56.5	0.9 (0.74, 1.1)	83.2	0.91 (0.52, 1.6)	84.2	1.54 (0.78, 3.01)
Sylhet	64.3	1.41 (1.15, 1.73)	62.9	1.55 (1.25, 1.92)	71.8	0.62 (0.36, 1.05)	74.3	0.75 (0.42, 1.34)
**Birth order**								
One	61.8	*Ref*	59.2	*Ref*	78.7	*Ref*	79.9	*Ref*
Two	61.8	**1.13 (1.01, 1.26)**	59.2	**1.13 (1.01, 1.27)**	79.7	1.18 (0.87, 1.61)	78.9	1 (0.71, 1.41)
Three	62.7	**1.3 (1.12, 1.5)**	60.7	**1.34 (1.15, 1.57)**	78.0	1.07 (0.71, 1.61)	77.6	1.02 (0.65, 1.6)
Four or more	61.1	**1.37 (1.15, 1.64)**	59.5	**1.48 (1.22, 1.78)**	72.8	0.88 (0.56, 1.4)	73.4	0.88 (0.52, 1.49)
**Religion**								
Others	61.5	*Ref*	59.3	*Ref*	83.5	*Ref*	83.6	*Ref*
Muslim	61.9	**1.2 (1.02, 1.41)**	59.5	**1.22 (1.03, 1.44)**	77.7	0.78 (0.46, 1.32)	78.0	0.77 (0.43, 1.38)
**Region**								
Rural	61.4	*Ref*	58.9	*Ref*	77.9	*Ref*	78.7	*Ref*
Urban	63.2	0.97 (0.88, 1.07)	60.9	0.97 (0.87, 1.07)	78.1	0.98 (0.73, 1.31)	77.2	0.79 (0.58, 1.09)
**ANC visits**								
No visit/Dk	47.1	*Ref*	43.6	*Ref*	67.9	*Ref*	69.5	*Ref*
1	50.0	1.04 (0.9, 1.21)	47.3	1.11 (0.94, 1.3)	69.2	0.97 (0.67, 1.4)	67.4	0.74 (0.49, 1.11)
2	62.0	**1.55 (1.34, 1.8)**	59.2	**1.62 (1.39, 1.9)**	82.0	**1.86 (1.28, 2.7)**	81.6	**1.54 (1.01, 2.37)**
3	64.6	**1.6 (1.38, 1.86)**	62.3	**1.7 (1.45, 2)**	79.1	**1.34 (0.91, 1.98)**	84.8	**1.75 (1.12, 2.75)**
4 or more	69.5	**1.87 (1.63, 2.15)**	67.6	**2.0 (1.72, 2.32)**	82.9	**1.49 (1.05, 2.11)**	81.1	1.2 (0.81, 1.78)
**Place of delivery**								
Home	50.8	*Ref*	47.6	*Ref*	69.5	*Ref*	70.3	*Ref*
Public	72.2	**2.39 (2.11, 2.71)**	69.4	**2.39 (2.09, 2.72)**	91.5	**4.51 (2.94, 6.91)**	83.4	**1.92 (1.33, 2.77)**
NGO	68.8	**2.08 (1.66, 2.61)**	66.9	**2.16 (1.7, 2.74)**	79.8	**2.06 (1.14, 3.72)**	81	**1.91 (1.01, 3.61)**
Private	71.1	**2.2 (1.99, 2.44)**	69.2	**2.29 (2.05, 2.55)**	86.6	**2.72 (2, 3.69)**	85.3	**2.22 (1.6, 3.08)**
**Mode of delivery**								
Single	61.8	*Ref*	59.4	*Ref*	78	*Ref*	78.3	*Ref*
Twin	67.4	1.06 (0.73, 1.53)	68.6	1.15 (0.78, 1.69)	79.1	1.46 (0.59, 3.65)	73.4	0.86 (0.28, 2.61)
